# Supplementary material for: Molecular Xenomonitoring (MX) allows real-time surveillance of West Nile and Usutu virus in mosquito populations
Source: PLoS Negl Trop Dis. 2024 Dec 26;18(12):e0012754. doi: 10.1371/journal.pntd.0012754 (PMC11709297; doi:10.1371/journal.pntd.0012754)
Supplement: S2 Table — Collection/Position field correspond to the first and second column of supplementary file 3 related to virus screening in individual mosquitoes. (DOCX) [file pntd.0012754.s002.docx]

|  |  |  |  | Excreta | | | Screening  moustiques individual | | | Vero  E6 cells | | | | | C6/36 cells | | | |
| --- | --- | --- | --- | --- | --- | --- | --- | --- | --- | --- | --- | --- | --- | --- | --- | --- | --- | --- |
| Isol. number | Mosquito species | Virus | Collection/Position | USUV Ct | WNV Duo Ct | WNV Linke Ct | USUV Ct | WNV Duo Ct | WNV Linke Ct | CPE | USUV Ct | WNV Duo Ct | WNV Linke Ct | dsRNA ELISA | CPE | USUV Ct | WNV Duo Ct | dsRNA ELISA |
| 1 | *Cx. pipiens* | WNV | C5/D6 | 33·2 | 26 | na | neg | 20·6 | 26·2 | + | neg | 11·4 | 13·4 | pos | + | neg | 16·3 | pos |
| 2 | *Cx. pipiens* | WNV | C1/G8 | neg | 19·8 | 20·1 | neg | 16·6 | 20·1 | + | neg | 11·1 | 12·5 | pos | + | neg | 13·9 | pos |
| 3 | *Cx. pipiens* | WNV | C1/H10 | neg | 19·8 | 20·1 | neg | 16·7 | 20·8 | + | neg | 11 | 12·7 | pos | + | neg | 13·1 | pos |
| 4 | *Cx. pipiens* | WNV | C7/D1 | 36·9 | 24·3 | 27·5 | neg | 15·2 | 18·5 | + | neg | 11·4 | na | pos | + | neg | 10.3 | pos |
| 5 | *Cs. longiareolata* | USUV | C6/B12 | 22·9 | 26·7 | neg | 17·2 | 20·4 | neg | + | 14·1 | 19·1 | neg | pos | + | 12·5 | 21 | pos |
| 6 | *Cx. pipiens* | USUV | C2/A1 | 27 | neg | na | 18·8 | 20·7 | neg | + | 12·8 | 17·3 | neg | pos | + | 12·1 | 16·2 | pos |
| 7 | *Cx. pipiens* | USUV | C2/D5 | 27 | neg | na | 28·6 | 31 | neg | + | 16·9 | 20·6 | neg | pos | - | neg | neg | pos |

**Supplementary table 2:** Ct values obtained from excreta, individual mosquito samples and Vero E6 and C6/36 supernatant samples obtained for isolated WNV and USUV strains. Collection/Position field correspond to the first and second column of supplementary file 3 related to virus screening in individual mosquitoes.
