# Supplementary material for: Molecular Xenomonitoring (MX) allows real-time surveillance of West Nile and Usutu virus in mosquito populations
Source: PLoS Negl Trop Dis. 2024 Dec 26;18(12):e0012754. doi: 10.1371/journal.pntd.0012754 (PMC11709297; doi:10.1371/journal.pntd.0012754)
Supplement: S3 Table — For each sequence, the exact source (excreta, mosquito, isolate), amplification approach, coverage at 30X (for sequences obtained from isolates) or 50X (for sequences obtained from mosquito and mosquito excreta samples), and Genbank accession number are specified. Sequencing reads for all virus genomes are available on NCBI (Bioproject ID: PRJNA1085973). Virus genomes with no Genbank accession number (NA*) are available at https://github.com/rklitting/WNV_USUV_NouvelleAquitaine_2023. (DOCX) [file pntd.0012754.s003.docx]

**Supplementary table 3:** WNV and USUV sequences produced from mosquito excreta and individual mosquitoes. For each sequence, the exact source (excreta, mosquito, isolate), amplification approach, coverage at 30X (for sequences obtained from isolates) or 50X (for sequences obtained from mosquito and mosquito excreta samples), and Genbank accession number are specified. Sequencing reads for all virus genomes are available on NCBI (Bioproject ID: PRJNA1085973). Virus genomes with no Genbank accession number (NA*) are available at https://github.com/rklitting/WNV_USUV_NouvelleAquitaine_2023.

| Sample ID | GB accession | Collection date | Location | Virus | Isolation source | Coverage |
| --- | --- | --- | --- | --- | --- | --- |
| C6/B12 | PP482814 | 2023-08-18 | Site M | USUV | VeroE6 (P0) | 61.02 |
| C6/B12 | PP482815 | 2023-08-18 | Site M | USUV | mosquito | 95.01 |
| C2/A1 | PP482816 | 2023-08-03 | Site E | USUV | mosquito | 89.62 |
| C2/A1 | NA* | 2023-08-03 | Site E | USUV | C636 (P0) | 40.28 |
| C2/D5 | PP482817 | 2023-08-03 | Site E | USUV | VeroE6 (P0) | 92.56 |
| C5/D6 | PP482818 | 2023-08-03 | Site F | WNV | C636 (P0) | 96.33 |
| C5/D6 | PP482819 | 2023-08-03 | Site F | WNV | VeroE6 (P0) | 98.23 |
| C5/D6 | PP482820 | 2023-08-03 | Site F | WNV | mosquito | 94.15 |
| C1/H10 | PP482821 | 2023-07-25 | Site E | WNV | VeroE6 (P0) | 89.26 |
| C1/H10 | PP482822 | 2023-07-25 | Site E | WNV | mosquito | 90.18 |
| C1/G8 | PP482823 | 2023-07-25 | Site E | WNV | VeroE6 (P0) | 92.29 |
| C1/G8 | PP482824 | 2023-07-25 | Site E | WNV | mosquito | 90.17 |
| C7/D1 | PP482825 | 2023-08-24 | Site K | WNV | C636 (P0) | 86.76 |
| C7/D1 | PP482826 | 2023-08-24 | Site K | WNV | mosquito | 90.17 |
| E1-M4 | NA* | 2023-08-03 | Site H | WNV | mosquito excreta | 88.08 |
| E10-M2 | NA* | 2023-07-25 | Site H | WNV | mosquito excreta | 5.04 |
| E11-M2 | NA* | 2023-07-25 | Site G | WNV | mosquito excreta | 11.99 |
| E15-M2 | NA* | 2023-07-27 | Site G | WNV | mosquito excreta | 83.02 |
| E2-M1 | NA* | 2023-07-25 | Site A | WNV | mosquito excreta | 36.51 |
| E2-M2 | NA* | 2023-07-25 | Site E | WNV | mosquito excreta | 93.59 |
| E2-M4 | NA* | 2023-08-03 | Site F | WNV | mosquito excreta | 84.83 |
| E6-M4 | NA* | 2023-08-03 | Site I | WNV | mosquito excreta | 86.57 |
| E4-M4 | NA* | 2023-08-03 | Site E | USUV | mosquito excreta | 18.92 |
