# Supplementary material for: Molecular Xenomonitoring (MX) allows real-time surveillance of West Nile and Usutu virus in mosquito populations
Source: PLoS Negl Trop Dis. 2024 Dec 26;18(12):e0012754. doi: 10.1371/journal.pntd.0012754 (PMC11709297; doi:10.1371/journal.pntd.0012754)
Supplement: S4 Table — (DOCX) [file pntd.0012754.s004.docx]

**Supplementary table 4: Primer sequences used to generate a non-overlapping amplicon ladder of different sizes to assess viral RNA degradation.**

| **Primers** | **Sequence 5'-3'** | **pb** |
| --- | --- | --- |
| AEFV_1_LEFT | TGAAGGTCACATCCCTAGTTGGA | 2948 |
| AEFV_1_RIGHT | AATGCATCGGGAGTGTATGACG |  |
| AEFV_2_LEFT | GTTCATGGTTCGACGCAAACTG | 1737 |
| AEFV_2_RIGHT | ATTGGGAAGTTTGAACCACCGT |  |
| AEFV_3_LEFT | GGGCTGTTATTCCATCCTGCTT | 924 |
| AEFV_3_RIGHT | TTCTTGTCCGCTCTTTGGTGAG |  |
| AEFV_4_LEFT | TGGGATACGAAGGTGAGTTCTGA | 524 |
| AEFV_4_RIGHT | TGGTAGTGATGTGAGCAAAATTCG |  |
| AEFV_5_LEFT | TCTGTTCGGAATTGGTGATGACC | 101 |
| AEFV_5_RIGHT | CCTAAGTCCAAAGTGTAAACATGGT |  |
