# Supplementary figures and images for: Molecular Xenomonitoring (MX) allows real-time surveillance of West Nile and Usutu virus in mosquito populations
Source: PLoS Negl Trop Dis. 2024 Dec 26;18(12):e0012754. doi: 10.1371/journal.pntd.0012754 (PMC11709297; doi:10.1371/journal.pntd.0012754)

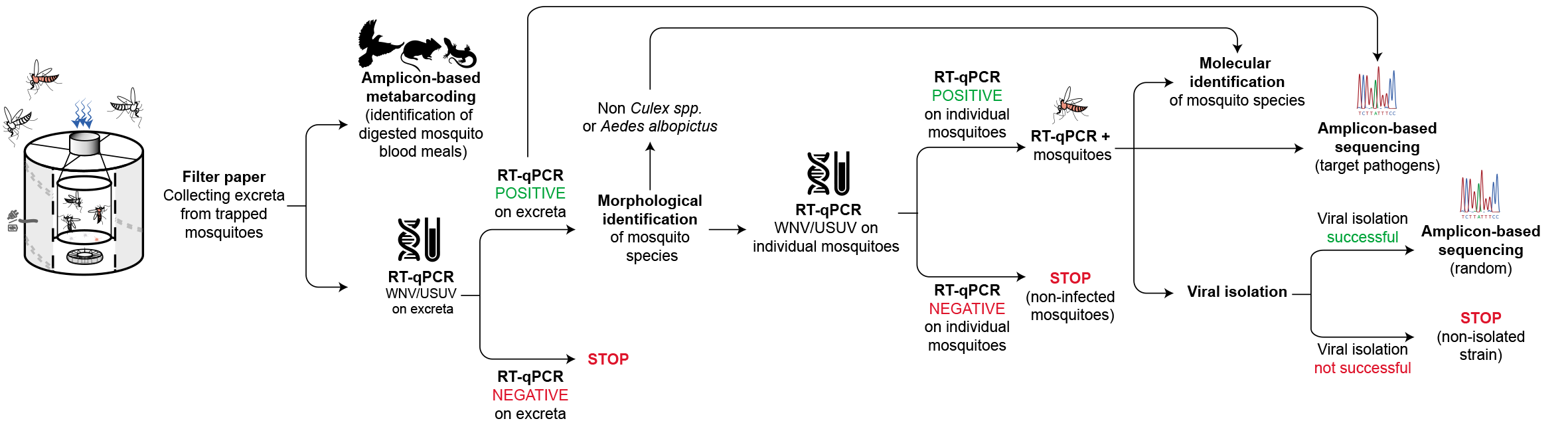

Supplement: S1 Fig — Mosquitoes are captured and kept alive on the field during several days in a 3D printed shelter with a free access to sugar water. Mosquitoes are then killed and kept frozen in situ while the filter papers containing their excreta are sent to a laboratory at room temperature by post. Virus detection is performed at first step directly on mosquito excreta by RT-qPCR. If positive, an attempt was made to sequence the genomic RNA of the virus using amplicon-based approaches directly on the excreta. Mosquitoes from collections found positive for either viruses were then transported to the laboratory on dry ice before to be analyzed individually. Estimation of infection rates in mosquitoes, virus isolation and sequencing were performed on trapped mosquitoes on a second step. (TIF) [file pntd.0012754.s005.tif]

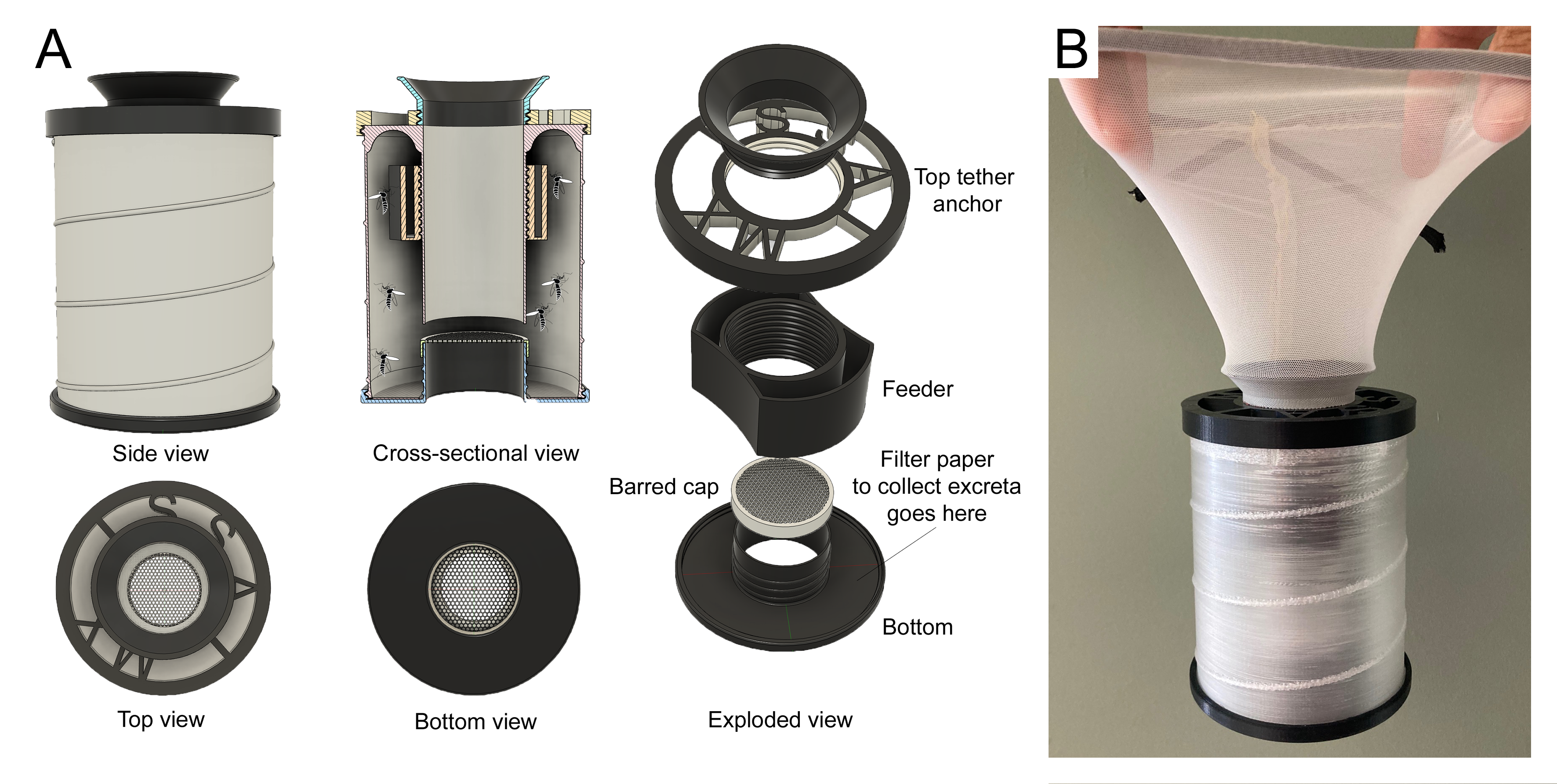

Supplement: S2 Fig — (A) Different views of the adapter. All components are visible in the cross-sectional and exploded views. (B) Picture of the adapter ready to be attached to the intake funnel of the BGS. The MX adapter was created on Fusion 360 (AutoDesk) and 3D printed in PLA. MX adapter 3D files (.stl format) are provided in S1 File under the Creative Commons (CC) license BY. (TIF) [file pntd.0012754.s006.tif]

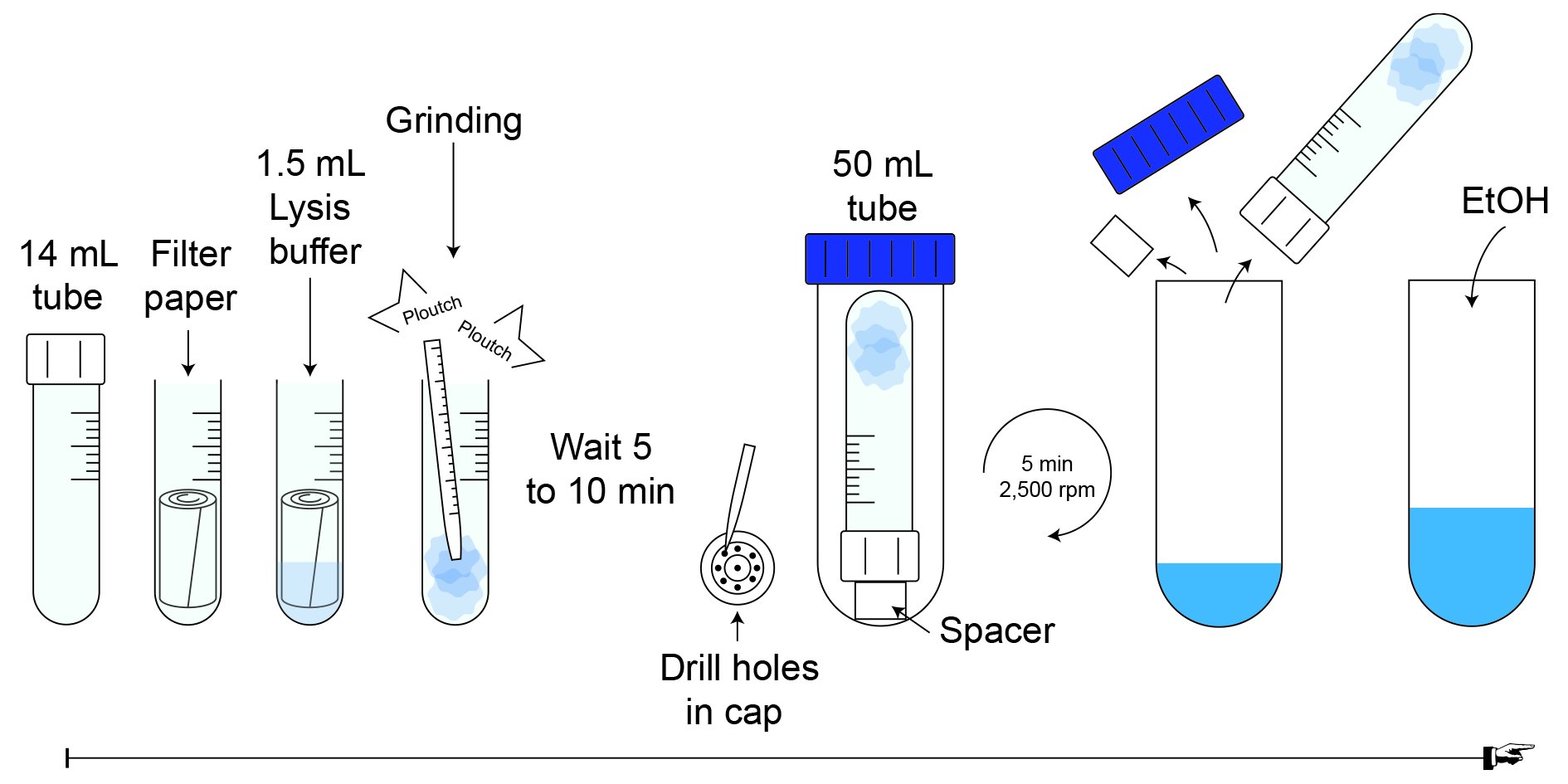

Supplement: S3 Fig — (TIF) [file pntd.0012754.s007.tif]

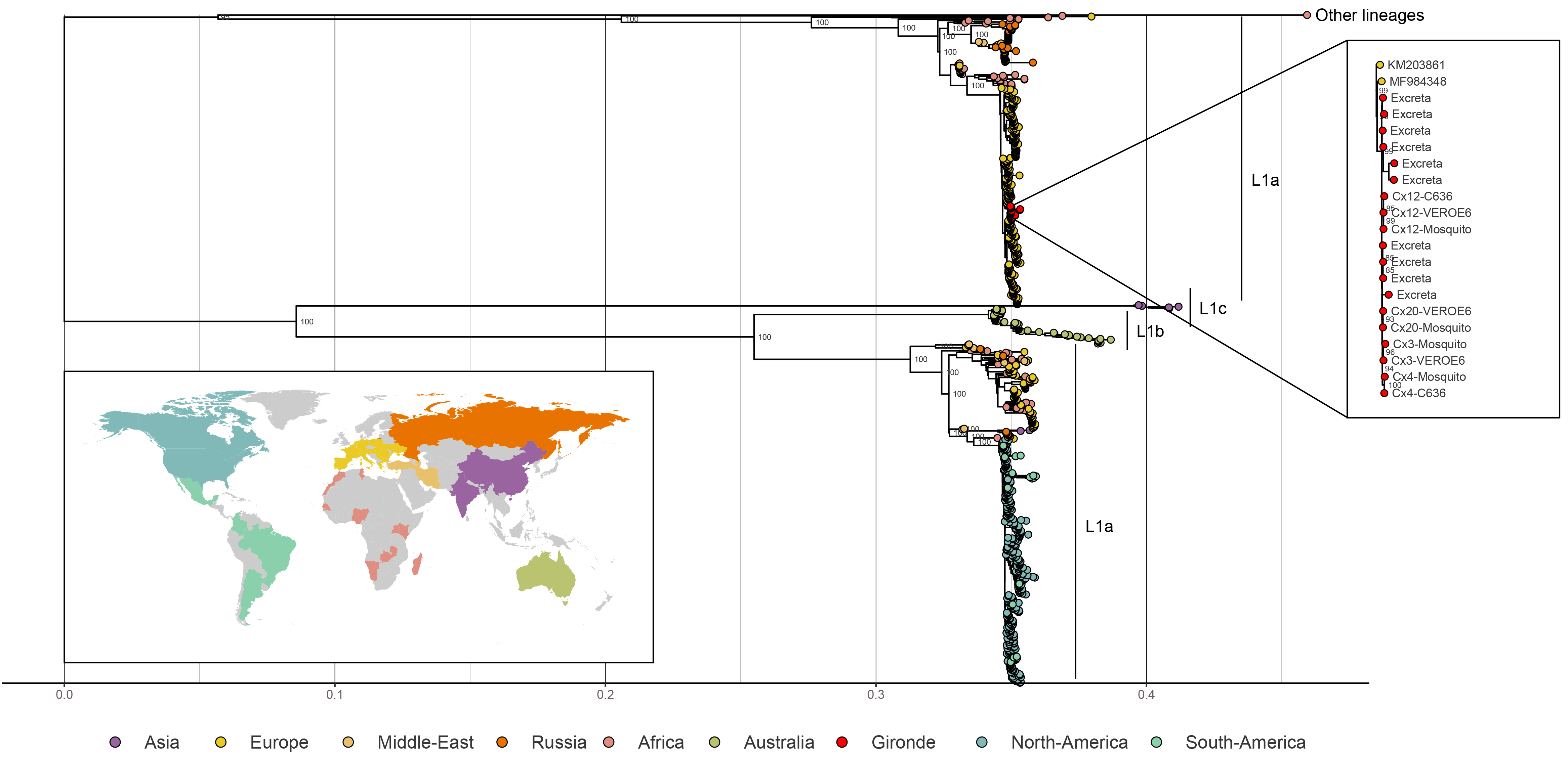

Supplement: S4 Fig — The Maximum-likelihood phylogeny was inferred using IQ-Tree under model finder. Branch support values were calculated using UFBoot (100 replicates). Statistical supports values superior to 80% are shown for the main clades. All sequences are colored according to their geographic origin. A zoom on the clade with WNV sequences from this work is shown on the right hand side of the panel (Excreta: sequences derived from mosquito excreta, VEROE6 and C636: sequences derived from VEROE6 and C636 cell cultures, respectively, Mosquito: sequences derived from single mosquitoes). Map base layers are from the R maps package available at CRAN: Package maps (r-project.org). (TIF) [file pntd.0012754.s008.tif]

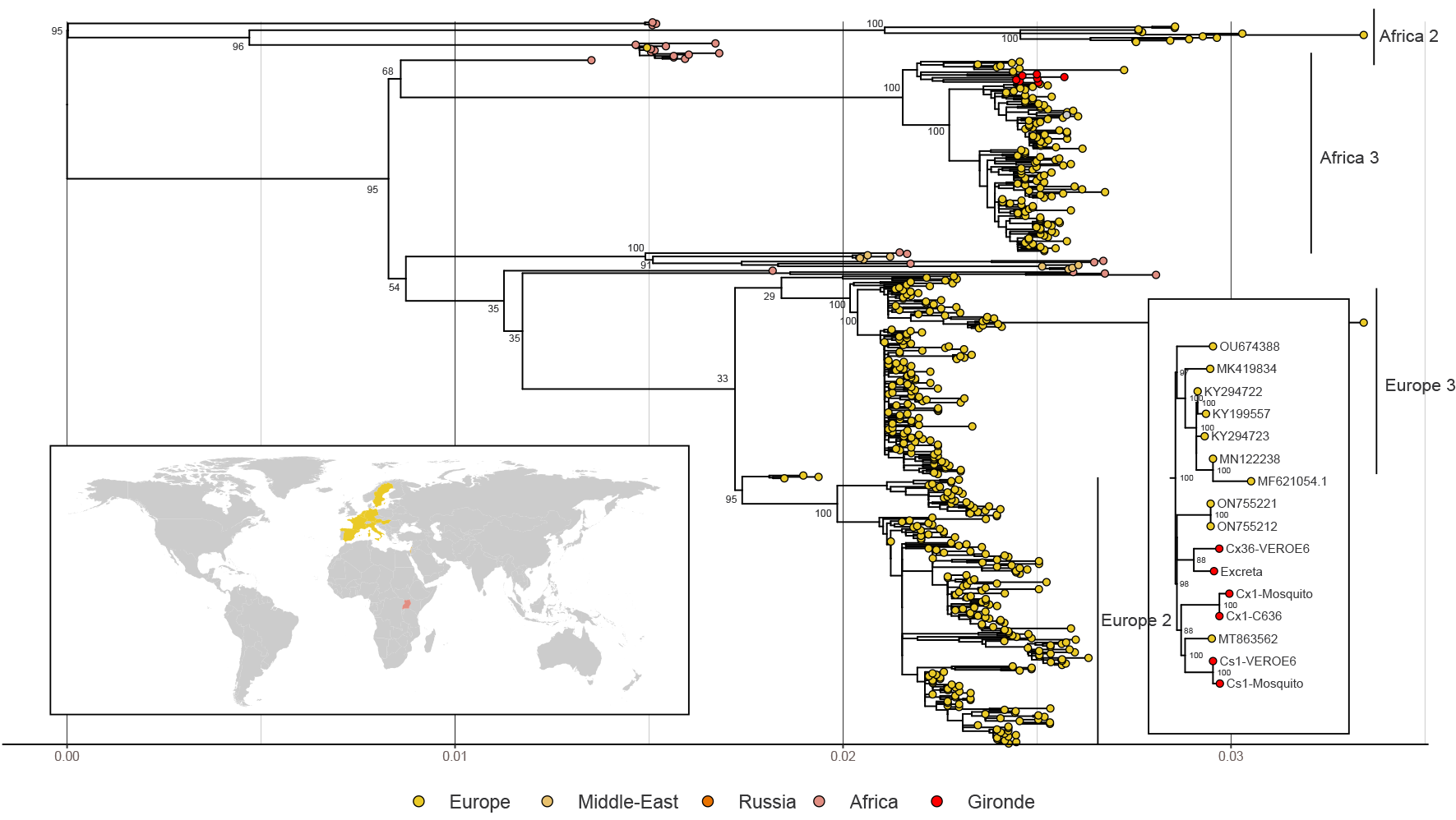

Supplement: S5 Fig — The Maximum-likelihood phylogeny was inferred using IQ-Tree under model finder. Branch support values were calculated using UFBoot (100 replicates). Statistical supports values superior to 80% are shown for the main clades. All sequences are coloured according to their geographic origin. A zoom on the clade with WNV sequences from this work is shown on the right hand side of the panel (Excreta: sequences derived from mosquito excreta, VEROE6 and C636: sequences derived from VEROE6 and C636 cell cultures, respectively, Mosquito: sequences derived from single mosquitoes). Map base layers are from the R maps package available at CRAN: Package maps (r-project.org). (TIF) [file pntd.0012754.s009.tif]

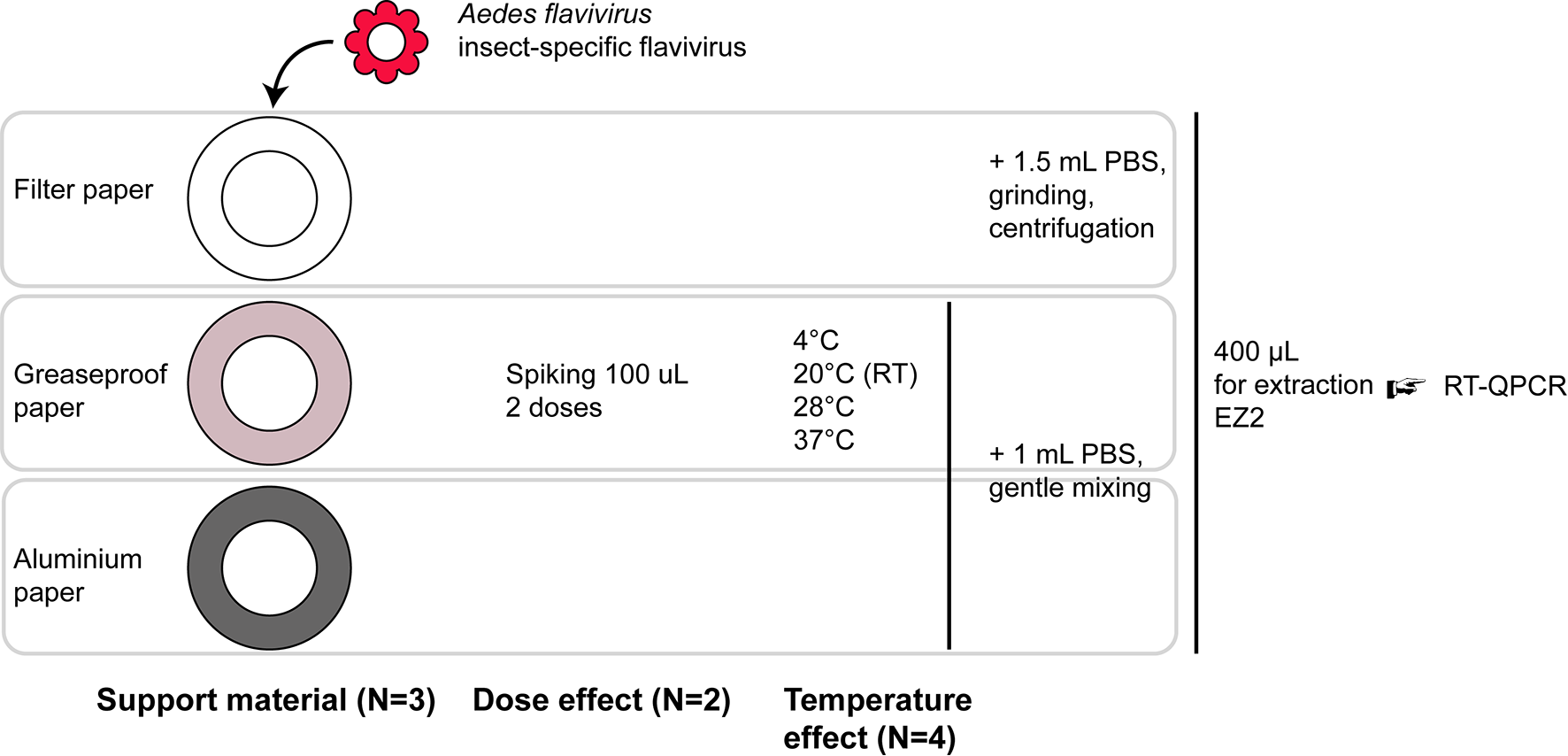

Supplement: S6 Fig — In this design, the volume of PBS was dependent of the support material. (TIF) [file pntd.0012754.s010.tif]

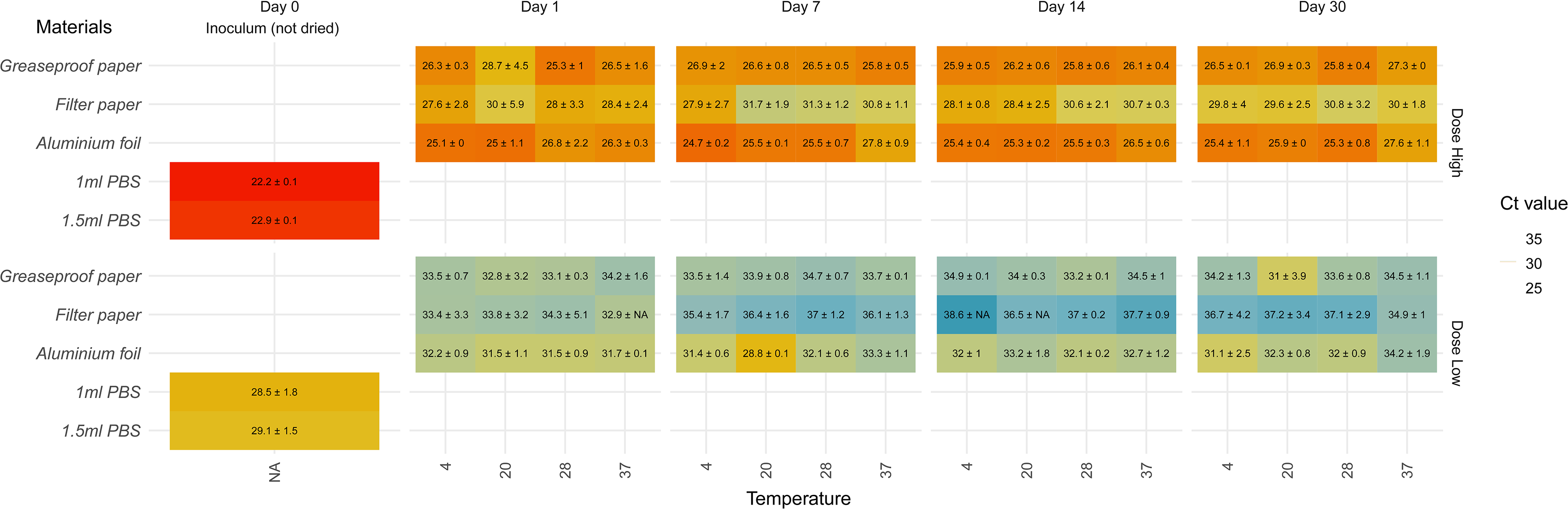

Supplement: S7 Fig — Mean (±SD) Ct values across replicates are presented in an heatmap. (TIF) [file pntd.0012754.s011.tif]

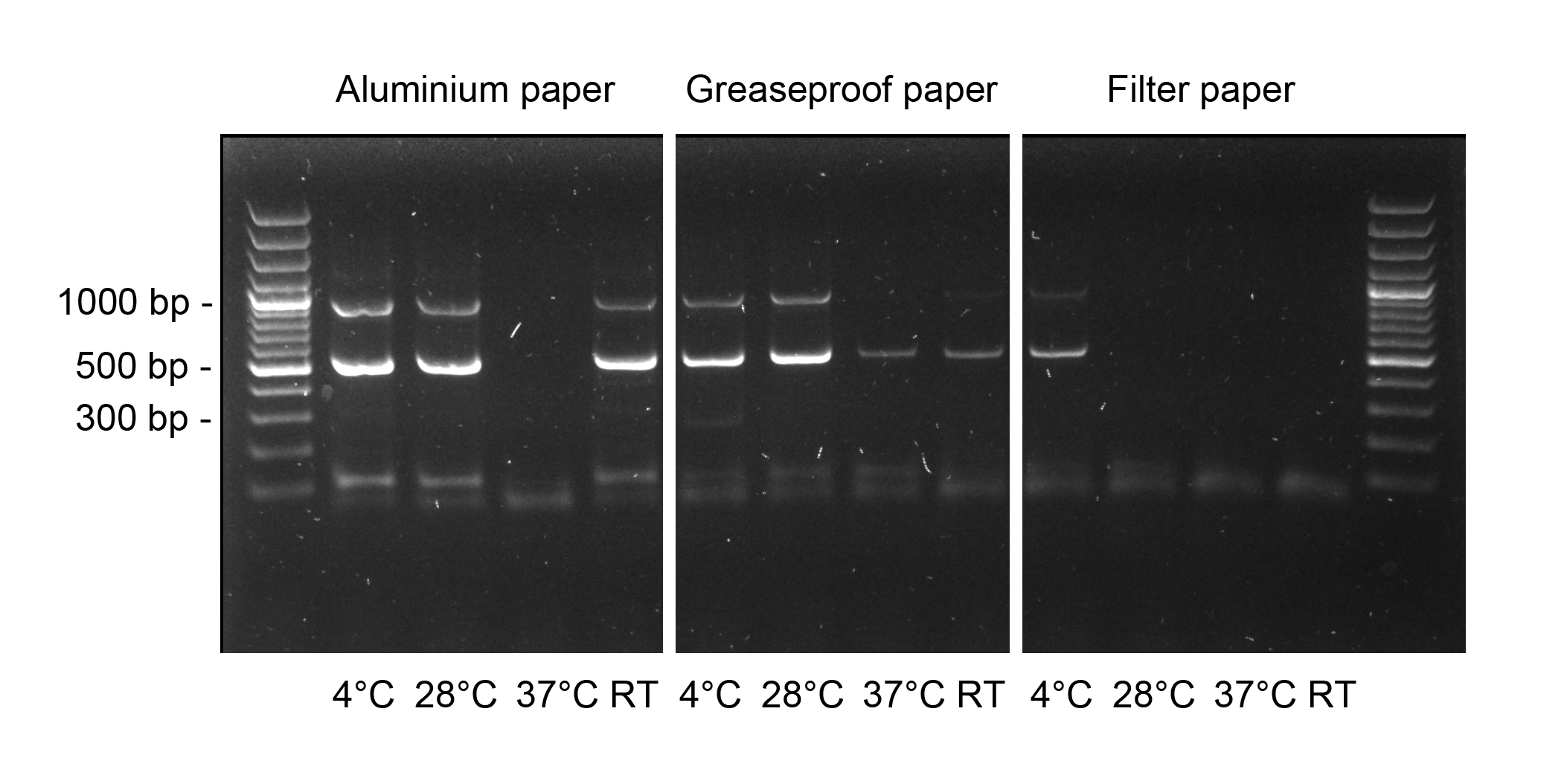

Supplement: S8 Fig — Viral RNA was exposed to various temperatures, support materials, and time points post-inoculation. Amplicons were visualized on a 1% agarose gel, providing insights into RNA integrity and stability across experimental conditions. (TIF) [file pntd.0012754.s012.tif]
