# Supplementary material for: Molecular Xenomonitoring (MX) allows real-time surveillance of West Nile and Usutu virus in mosquito populations
Source: PLoS Negl Trop Dis. 2024 Dec 26;18(12):e0012754. doi: 10.1371/journal.pntd.0012754 (PMC11709297; doi:10.1371/journal.pntd.0012754)
Supplement: S4 File — This supplementary file present results of a spiking and recovery experiment that aim to assess how different transportation and storage conditions impact the molecular detection sensitivity of Flavivirus RNA. The materials and methods, as well as the results and discussion, are provided in this file. (DOCX) [file pntd.0012754.s016.docx]

## Evaluation of molecular detection sensitivity in preserved dried *Flavivirus* RNA: effects of time, temperature, and virus Dose.

## Objectives:

## The objective of this study is to assess how different transportation and storage conditions impact the molecular detection sensitivity of *Flavivirus* RNA.

## Material and method:

### Virus isolation and production

*Aedes flavivirus* (AEFV) isolated from an individual *Aedes albopictus* female in Marseille in 2021 was used in this experiment. AEFV is an insect specific *flavivirus* with the similar morphologic and genomic characteristics than other dual-host arboviruses from the same *Orthoflavivirus* family, such as West Nile virus (WNV). AEFV is however noninfectious to human which facilitate its manipulation in standard laboratory. The virus had been passaged twice in C6/36 cells prior to use in the experiments, with the last passage implemented during the month before the experiment (February 2024).

### Preparation of *In Vitro* RNA transcripts

Standard stock solution of AEFV RNA transcripts was prepared by amplifying a 924 bp segment from virus isolate cDNA, which includes the detection target, using a forward primer with a T7 promoter sequence attached at its 5’ end (5'-TAATACGACTCACTATAGGGAGAAGGGTGGGATACGAAGGTGA-3’) and a reverse primer (5'-GCGATGTCTTGCCTCTTGTGTA-3’). Purified AEFV RNA was first reverse transcribed to complementary DNAs (cDNAs) with random hexamers using M-MLV reverse transcriptase (Life Technologies) according to the manufacturer's instructions. Amplifications were performed with Phusion Green Hot Start II High-Fidelity PCR Master Mix (Thermo Scientific) using the following program: polymerase activation at 98 °C for 5 min, followed by 35 PCR cycles of 30 sec at 98 °C, 30 sec at 60 °C and 1 min of elongation at 72°C with 7 min of final elongation at the end of the cycles. Amplicons were purified using AMPure magnetic beads with a 1:1 ratio as per the manufacturer’s protocol (two washes in 80% ethanol and final elution in water) and quantified using the Qubit DNA HS assay. Transcription was carried out following the MAXIscript kit protocol (MaxiScript SP6/T7 kit, Ambion, France), using 12 μL of purified amplicons with 1 μL of each nucleotide and 2 μL of T7 Enzyme Mix. Reactions were incubated at 37°C for 1 hour. Following this, 1 μL of Turbo DNase was added to each reaction, with further incubation at 37°C for 15 minutes to digest the cDNA template post-transcription. To inactivate DNase, 1 μL of 0.5 M EDTA was added, followed by incubation at 90°C for 5 minutes. RNA was then purified with AMPure magnetic beads, and quantified using the Qubit RNA HS assay after a 1/10 dilution. The RNA molecule copy number was calculated following the manufacturer’s instructions (MaxiScript SP6/T7 kit) using the following formula: *Y* molecules/*µ*L = (*X* g/*µ*L RNA/[transcript length in nucleotides × 330] × 6.022·10^23^). The estimated concentration was 5 x 10^9^ copies per μL. In Vitro RNA transcripts were aliquoted, and stored at -80°C.

### Virus titration and detection

### AEFV detection was conducted using a one-step reverse transcription quantitative polymerase chain reaction (RT-qPCR) assay, targeting a 150 bp region within the NS5 protein-coding gene. The assay utilized the One-Step TB Green® PrimeScript™ RT-PCR Kit II (Perfect Real Time, Takara, Dalian, China) and was prepared with a reaction mix containing 1x buffer, 200 nM of PCR primers (Forward: 5'-CCGGAACATAGTGGCGATGT-3’; Reverse: 5'-GCCACTTGGACTTTACCGTTC-3'). Amplification was carried out under the following thermal cycling conditions: reverse transcription at 42 °C for 5 minutes, followed by polymerase activation at 95 °C for 10 seconds, and then 40 PCR cycles of 5 seconds denaturation at 95 °C and 20 seconds of annealing and extension at 60 °C. The fluorescence signals were recorded at the end of each cycle. Absolute quantification was achieved by comparing cycle threshold values to a standard curve generated from a series of 10-fold dilutions of known concentrations. Virus detection from samples was performed without absolute quantification. All amplifications were executed on a CFX real-time thermocycler (Bio-Rad).

### Generation of an amplicon ladder to assess RNA degradation by end-point PCR.

A multiplex assays of 5 non-overlapping primers of different sizes was developed to assess viral RNA fragmentation and degradation by end-point PCR. Virus genomic RNA was first reverse transcribed to complementary DNA (cDNA) with random hexamers using M-MLV reverse transcriptase (Life Technologies, Inc.) according to the manufacturer's instructions. For the multiplex PCR reactions, 5 μl of cDNA was used in a 20 μl reaction mixture made of 5 μl of Hot START 5X BIOamp DNA polymerase mix (Biofidal), 4 μl of forward and reverse primer mix at 10 μM, and 11 μl of water. The thermal programme was: 10 min of polymerase activation at 96°C followed by 35 cycles of (i) 30 s denaturing at 96°C, (ii) 30 s annealing at 60°C and (iii) 1 min extension at 72°C, followed by a final incubation step at 72°C for 7 min to complete synthesis of all PCR products. Amplicons were visualized by electrophoresis on a 1% agarose gel.

| **Primers** | **Sequence 5'-3'** | **pb** |
| --- | --- | --- |
| AEFV_1_LEFT | TGAAGGTCACATCCCTAGTTGGA | 2948 |
| AEFV_1_RIGHT | AATGCATCGGGAGTGTATGACG |  |
| AEFV_2_LEFT | GTTCATGGTTCGACGCAAACTG | 1737 |
| AEFV_2_RIGHT | ATTGGGAAGTTTGAACCACCGT |  |
| AEFV_3_LEFT | GGGCTGTTATTCCATCCTGCTT | 924 |
| AEFV_3_RIGHT | TTCTTGTCCGCTCTTTGGTGAG |  |
| AEFV_4_LEFT | TGGGATACGAAGGTGAGTTCTGA | 524 |
| AEFV_4_RIGHT | TGGTAGTGATGTGAGCAAAATTCG |  |
| AEFV_5_LEFT | TCTGTTCGGAATTGGTGATGACC | 101 |
| AEFV_5_RIGHT | CCTAAGTCCAAAGTGTAAACATGGT |  |

Table 1: Primer sequences used to generate a non-overlapping amplicon ladder of different sizes to assess viral RNA degradation.

### Experimental design

### Virus preservation was evaluated based on four parameters: the effect of support material (3 types: filter paper, greaseproof paper, and aluminum foil), the effect of virus dose (2 levels: low dose with 3 x 10⁶ RNA copies in 100 μl of deposited supernatant, and high dose with 3 x 10⁸ RNA copies in 100 μl of deposited supernatant), temperature (4 temperatures: 4°C, ambient temperature (around 20°C), 28°C, and 37°C), and time post-inoculation (4 time points: Day 1, Day 7, Day 14, and Day 30). The criteria of cost and accessibility informed the selection of support materials. Importantly, greaseproof paper, and aluminum foil has less capacity to retain liquid than filter paper and allows the virus to be rinsed without the need for centrifugation.

### For each virus dose condition, 100 μl of virus was applied to the support material and stored at each temperature until sample collection. During collection, 1 mL of PBS was added to the greaseproof paper and aluminum foil supports, which had been rolled into a 12 mL tube. The samples were then vortexed for 5 minutes to elute the dried virus inoculum. For filter paper, 1.5 mL of PBS was added to ensure full recovery of the sample due to the paper’s higher liquid absorption capacity. The PBS containing the eluted virus was recovered as specified in the main protocol for the filter paper condition, and directly recovered by pipetting in the tube for both other support materials. 400 μl of PBS were used for RNA extraction using the EZ2 automation system (Qiagen) according to the manufacturer’s instructions. To reduce potential handling errors and biases, this experiment was performed in duplicate. At the final step, total RNA from each sample was eluted in 60 μL of RNase-free water


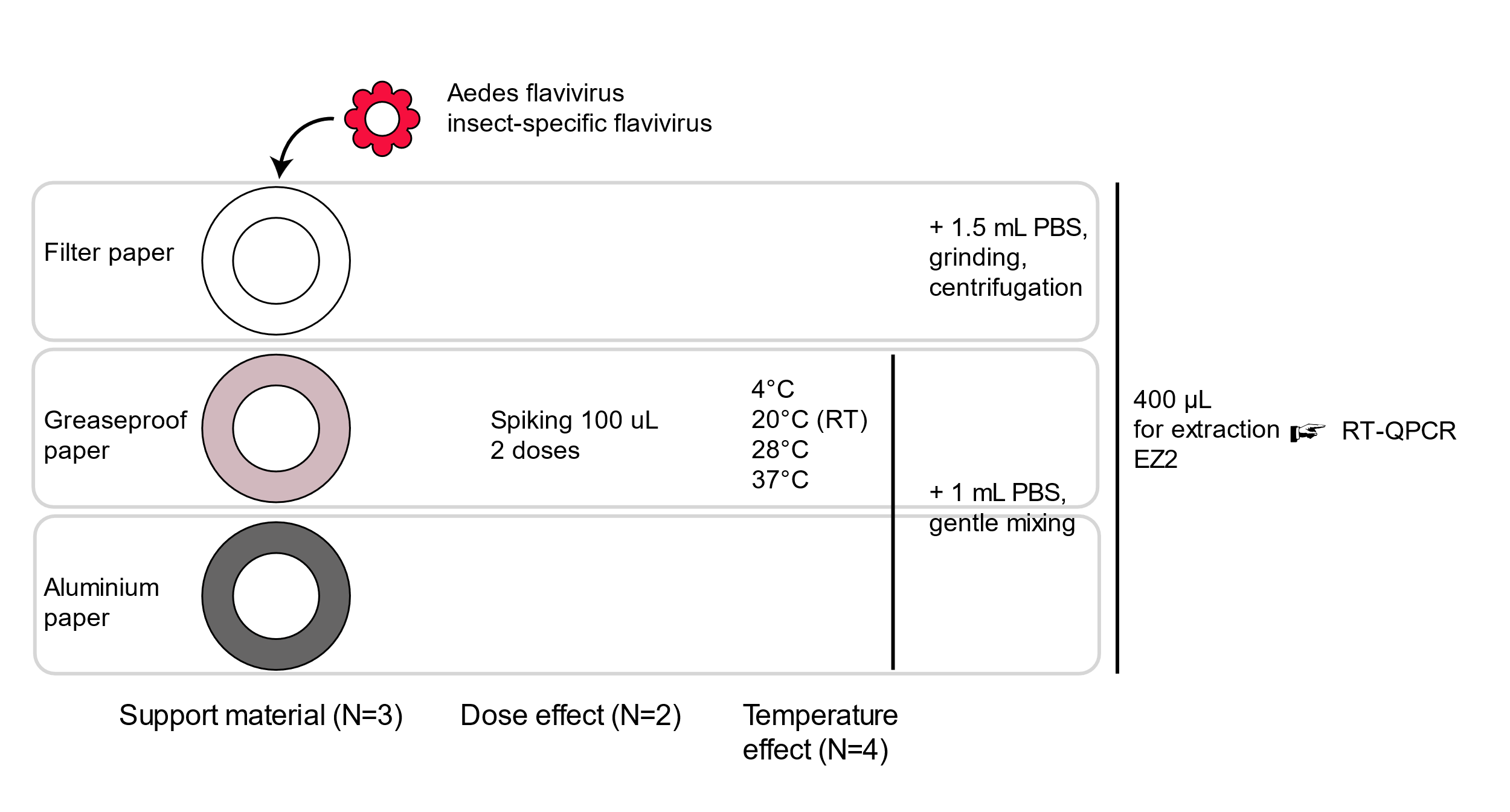


### Figure 1: Schematic representation of the procedure used to assess the Molecular Detection Sensitivity in Preserved Dried *Flavivirus* RNA. In this design, the volume of PBS was dependent of the support material.

### Statistical analyses

An ANOVA model was employed to evaluate the impact of each parameter on virus preservation, with cycle threshold (Ct) values serving as the readout. The model included factors such as support material, virus dose, temperature, sampling Time (in days), and replicate with interactions. The emmeans method was used as *post hoc* test to obtain estimated marginal means and contrasts across pairwise factor comparisons. The analysis was conducted using the rstatix package in R.

## Results

An average reduction of 5 cycle threshold (Ct) values was observed when comparing detection from direct virus inoculum (undried) to dried virus inoculum, independent of virus dose, support material, temperature, or time post-inoculation. Virus RNA was detected under all conditions throughout the study. The amount of virus detected was significantly influenced by both virus dose (p = 3.91e-46) and support material (p = 2.76e-22). Notably, detection was significantly lower with filter paper compared to other support materials (Bonferroni-adjusted p < 0.01 for each pairwise comparison involving filter paper). This reduced detection may be due, at least in part, to the larger PBS volume required for rinsing filter paper, which increased the dilution factor. Additionally, a significant interaction was noted between support material and replicate (p = 1.06e-01), indicating that the effect of support material varied slightly across replicates. No significant effect of time post-inoculation was observed across all temperatures. AEFV inoculum remained detectable by RT-qPCR even after one month at 37°C.

##
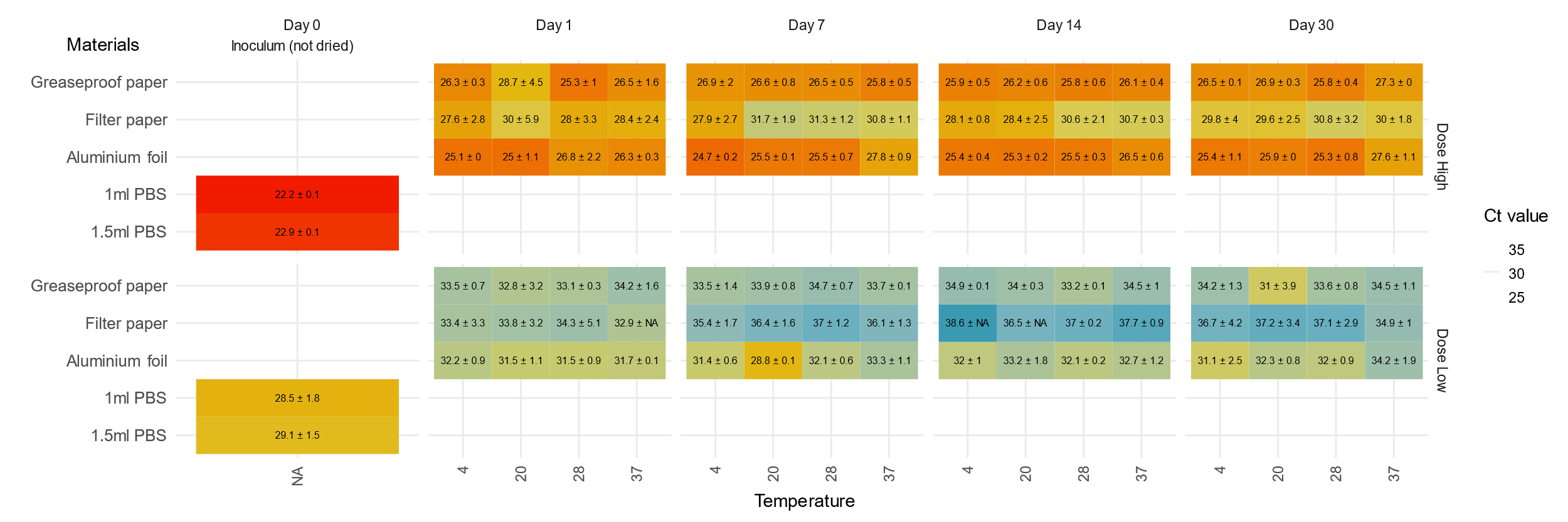


**Figure 2: Virus detection as cycle threshold (Ct) based on time post-inoculation, temperature, and support material.** Mean (±SD) Ct values across replicates are presented in an heatmap.

RT-qPCR quantifies viral load by detecting short target sequences (here 150 base pairs). However, this method may not accurately reflect viral RNA degradation, as fragmented viral RNA can still contain short intact sequences that are amplifiable by RT-qPCR. In contrast, determining the genetic identity of viruses through sequencing requires the amplification of larger genomic sections, typically ranging from 500 to 1000 base pairs. Our experiments showed that viral RNA fragments up to 1000 base pairs could be successfully amplified from all tested materials after one month of storage at 4°C. However, on filter papers stored at temperatures above 4°C, PCR amplification of fragments larger than 100 base pairs failed. Notably, larger amplicons (up to 1000 base pairs) were successfully generated on aluminum foil and greaseproof paper stored at temperatures between 4°C and 28°C, even after one month of virus inoculation.
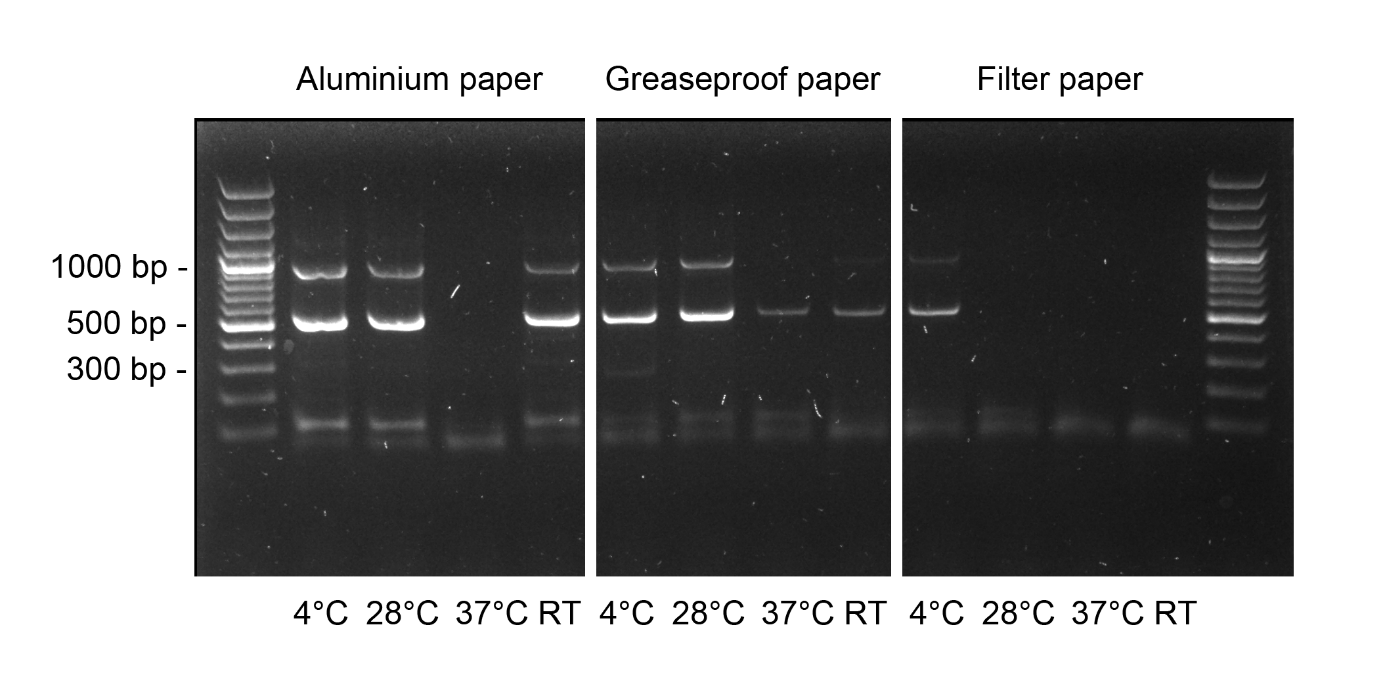


**Figure 3: Evaluation of viral genomic RNA fragmentation at 30 days post virus inoculation as a function of temperature and support material by using a ladder of non-overlapping amplicons.** RT: Room temperature.

## Conclusions

*Flavivirus* RNA remained detectable by RT-qPCR across all tested conditions, including varied support materials, virus doses, temperatures, and durations up to one month, even at 37°C. This demonstrates the robust preservation of viral RNA, at the dried state, under diverse storage conditions. Filter paper yielded lower detection levels compared to greaseproof paper and aluminum foil, likely due to the higher dilution factor resulting from the greater PBS volume required for rinsing filter paper. Support materials with low liquid retention capabilities are preferable for collecting and preserving virus RNA, as they simplify the extraction process by eliminating the need for centrifugation. "Furthermore, viral genomic integrity appears to be better preserved on these materials. Amplicon tiling methods can be successfully applied to cDNA generated from purified RNA extracted from mosquito excreta, even after several weeks of transport without the need for a cold chain. To maximize RNA preservation, we still recommend storing samples at the lowest possible temperature (not freeze to maintain membrane and capsid integrity). These findings offer valuable insights into optimal storage and transport conditions for *flavivirus* RNA, with practical implications for field sampling and laboratory processing, particularly in resource-limited settings or when transport over long distances is required.
