# Supplementary material for: Molecular Xenomonitoring (MX) allows real-time surveillance of West Nile and Usutu virus in mosquito populations
Source: PLoS Negl Trop Dis. 2024 Dec 26;18(12):e0012754. doi: 10.1371/journal.pntd.0012754 (PMC11709297; doi:10.1371/journal.pntd.0012754)
Supplement: Attachment — Submitted filename: Response to reviewers_PNTD.docx [file pntd.0012754.s017.docx]

Editor Comments to Authors:

**1.**The reviewers provided suggestions on improving minor issues with the manuscript (writing, typos, grammar, etc). The main critique was shared by multiple reviewers and the authors should adapt their manuscript accordingly in response to this critique. Namely, the authors should relate their "new" method explicitly to "older" established methods. This could be accomplished by experimentation (suggested by one reviewer, but not necessary, in the opinion of the editor). As suggested by some reviewers, this ideally would be addressed by including more discussion of the results of the current manuscript relative to (1) prior manuscripts on the topic of xenomonitoring and (2) more classical methods of vector surveillance. In other words, make a stronger case for xenomonitoring by explicitly comparing it to classical methods, including direct comparison of the observed results to expectations based on classical surveillance and previous findings of xenomonitoring. This was in the manuscript already to a limited extent, but the reviewers request a more obvious/stronger comparison.

**Response:** *We thank the reviewers and editor for their positive feedback. Additional insights on the origins and evolution of MX have been included in the discussion section. The standard entomological surveillance methods used to detect WNV in mosquitoes are now outlined in the introduction, along with typical infection rates reported in various studies. We also propose adding Supplementary File 4, which presents results from a 'spiking and recovery' experiment, demonstrating that viral RNA can be preserved for over a month at various storage temperatures and detected by RT-qPCR. Furthermore, the experiment shows that it is feasible to generate large amplicons from samples collected and transported at room temperature for several days before processing. Lastly, a section discussing the limitations of MX has been added to the discussion. Detailed responses and corresponding modifications can be found following each reviewer’s comment.*

**Line 130: “**Detecting arboviruses in field-collected mosquitoes is primarily used worldwide as an early warning system for viral circulation and to assess the risk of human transmission [20] . In most cases, arbovirus surveillance involves pooling mosquitoes by species, date, and location before extracting and detecting viral genomic RNA. For WNV surveillance, thousands to hundreds of thousands of mosquitoes are typically collected over a season, leading to pools of 50 to 200 mosquitoes per sample to minimize costs, with reported minimum infection rates ranging from less than 2% [21–24] to 10% [24,25] across studies. ”

**Line 488: “**Xenomonitoring broadly refers to the detection of vector-borne human pathogens in blood-feeding arthropod vectors. As the name suggests, it enables ongoing surveillance of pathogens in the environment without the need for human testing. One of the earliest formal applications of xenomonitoring was for lymphatic filariasis (LF), where it helped health programs assess the success of mass drug administration (MDA) campaigns aimed at eliminating the disease [47]. Initially reliant on observing parasites in dissected insects under a microscope, the concept evolved into Molecular Xenomonitoring (MX) with the advent of molecular detection methods, which enhanced sensitivity and improved large-scale screening by detecting pathogens at the species level in pools of captured arthropods [48]. MX is now used for a range of mosquito-borne parasites and viruses , as well as for pathogens transmitted by other vectors [47]. Here, we extend the noninvasive concept of MX (i.e., external molecular surveillance) by detecting viruses directly in the excrement of mosquitoes collected in the field.”

**Line 596:** Limitations. In this study, as in previous one [28], viral RNA was detected in the excreta of trapped mosquitoes, even when none was found in the mosquitoes themselves. Although we cannot completely rule out the possibility of mosquitoes escaping during sampling or trap handling, the more plausible explanation is the presence of predatory arthropods, such as ants and spiders, using the traps as food storage. Ants, in particular, probably first attracted by the sugar source inside the adapters, have been observed severing mosquitoes' legs or wings and carrying them back to their nests, leaving detached mosquito parts in the collections. This recurring issue, particularly associated with long-term field captures over several days, remains difficult to resolve. Integrated approaches that leverage site-specific solutions can help mitigate this issue, such as suspending traps from a glue-covered wire or placing them over a basin of water.

Excreta-based contamination of viral RNA on the body surface of mosquitoes trapped alongside infected individuals has been observed in other organisms [57], suggesting this possibility cannot be ruled out here. Such contamination could lead to an overestimation of arbovirus prevalence using the MX method and may complicate the identification of mosquito vector species involved in local transmission. Determining the vector competence of mosquitoes in the field is challenging, regardless of the trapping method used. While dissecting individual mosquitoes can provide insights, it is impractical for large-scale screening. Field studies have typically reported WNV infection rates in Culex mosquitoes ranging from 0.05% to 8%, depending on factors such as location, environmental conditions, and proximity to local bird reservoirs. [58,59]. Our estimate falls within this typical range, indicating consistency with broader findings on WNV prevalence in mosquito populations. High viral loads detected in individual mosquito homogenates can help identify the mosquito vector species responsible for local transmission. While more field-based observations are essential for understanding the role of local mosquito species in WNV or USUV transmission, vector competence assays in controlled experimental settings can further clarify this issue [60,61]. ”

## Reviewer Comments to Authors:

**Reviewer #4:**

**2.**The authors could have tried to think of some limitations and given the claims that the method is highly resource and cost effective, it would have been nice to see some numbers, however roughly generated, income prison with alternative approaches.

**Response:** *One major advantage of the MX strategy is that its costs and processing time are independent of the number of trapped mosquitoes. By targeting the excreta shed by an entire community of trapped mosquitoes, only a single RNA extraction and a single Q-RT-PCR reaction are required, whether for one or hundreds of mosquitoes. Consequently, cost reduction is directly tied to the number of mosquitoes collected over the study period.*

*It is challenging to provide a precise cost estimate, as savings would also depend on the reference strategy being used. For example, pooling mosquitoes by species prior to extraction is a common practice to reduce costs, with prices varying based on pool size. Here is a theoretical cost reduction analysis based on the 364 mosquitoes collected from seven sample collections (Table 1). Assuming the cost of an extraction and Q-RT-PCR detection is 5 euros, the total costs would be as follows:*

- ***1,820 euros*** *for individual detection (which was ultimately performed in this study).*
- ***180 euros*** *for a strategy involving the pooling of 10 mosquitoes per sample, representing a* ***10-fold cost reduction.***
- ***35 euros*** *(5 euros × 7 excreta samples) with the MX strategy, representing a* ***52-fold cost reduction.***

*It is worth highlighting that MX preserve trapped mosquitoes, it is a scalable strategy. Depending on their objectives, users can choose to process the trapped mosquitoes or not, thereby gaining additional information with corresponding additional costs. At last, MX limitations are now indicated in the discussion section (refer to question 4 and 17).*

**Reviewer #2:**

**3.**Line 77-84: can be deleted. Line 70-76 and 77-84 repeat the same thing.

**Response:** *Please accept our apologies for this error. This redundance is now deleted.*

**4.** Line 383-384: it would be better to write the genus in full for all species (*Ochlerotatus caspius*, *Ades vexans*, *Culiseta longiareolata* and *Aedes albopictus*). Line 386: *Ochlerotatus caspius* and *Culiseta longiareolata* can be shortened in *Oc. caspius* and *Cs. Longiareolata.*

**Response:** *Thank you, the changes have been made as requested.*

**5.**Line 514-518: A total of 19/364 mosquito specimens positive to WNV are reported in this study (*Cx. pipiens* N=14, *Ochlerotatus caspius* N=2, *Aedes vexans* N=2, *Aedes albopictus* N=1). More than one fourth of the positive sample is represented by species occasionally found in nature positive for this virus, with unclear or unproven vector role (e.g. *Ochlerotatus caspius*, *Aedes vexans*, *Aedes albopictus*). According to the authors, it is plausible that such positivity in non-vector species could be determined by a blood meal on viraemic hosts without being infected. This finding could be explained by high viral circulation in their hosts and is also suggested by the high number of positive excreta samples (39/52). In my opinion, positivity in mosquitoes should be better investigated by extending the analysis to a larger number of samples to strengthen the finding of high viral circulation (at least in their vectors) and exclude contamination in non-vector species.

**Response:** *The reviewer raised a pertinent question regarding mosquito positivity rates and the discrimination of vector species. Indeed, molecular detection of viruses in individual mosquito homogenates, following an extraction step, cannot elucidate the specific vector roles of each species. A mosquito might test positive because (i) it became infected after feeding on a viremic host, possibly amplifying the virus, (ii) it ingested a viremic blood meal but could not establish infection, or (iii) it was contaminated by excreta from another positive mosquito in the trap. At this stage, it is impossible to exclude any of these possibilities.*

*Firstly, excreta-based contamination has been reported by other researchers in different organisms [1], indicating that this possibility cannot be ruled out here. While we believe that the likelihood of oral infection via sugar meals in captivity is low due to the lack of a replication in intermediate host, contamination may occur through the deposition of shed virus particles or viral RNA on the body surface of mosquitoes trapped alongside an infected individual. Experimental designs in insectary facilities would be valuable for further exploration. Specifically, studies could investigate the occurrence of contamination by excreta, factors influencing the probability of contamination such as viral load and captivity duration, and potential preventive procedures, like thoroughly rinsing mosquitoes before homogenization.*

*Secondly, differentiating between various states—such as infectious, infected but non-infectious (and potentially never infectious), newly infected but not yet infectious, or non-infected but fully or partially engorged with undigested or partially digested infectious blood—is complex. Importantly this challenge, which involves disentangling the vector competence status of trapped mosquitoes, remains irrespective of the trapping method employed. Organ dissection of each trapped mosquito can provide answers, but it is definitively not suitable for large-scale screening.*

*Finally, 19/364 represent 5% of positivity rate. As specified in the paper, these results may overestimate positivity rates, as we only tested mosquitoes from traps for which a PCR-positive result was obtained for WNV or USUV in the corresponding excreta. Research typically finds WNV infection rates in Culex mosquitoes to range from 0.05% to 8% using detection in pools, with variations based on the location, environmental conditions or proximity with local bird reservoir [2,3]. Our estimate falls within this typical range, indicating consistency with broader findings on WNV prevalence in mosquito populations.*

*In conclusion, we strongly agree with the reviewer that more research is needed to fully understand the level of information that the surveillance method provides about the vector competence of trapped mosquitoes. While more field-based observations will help, controlled experimental designs would be more helpful in providing accurate answers. The virus load detected in each mosquito and the prevalence by species can be however a good proxy for the vector species involved. This is now summarized in the discussion section:*

**Line 606: “**Excreta-based contamination of viral RNA on the body surface of mosquitoes trapped alongside infected individuals has been observed in other organisms [48], suggesting this possibility cannot be ruled out here. Such contamination could lead to an overestimation of arbovirus prevalence using the MX method and may complicate the identification of mosquito vector species involved in local transmission. Determining the vector competence of mosquitoes in the field is challenging, regardless of the trapping method used. While dissecting individual mosquitoes can provide insights, it is impractical for large-scale screening. Field studies have typically reported WNV infection rates in Culex mosquitoes ranging from 0.05% to 8%, depending on factors such as location, environmental conditions, and proximity to local bird reservoirs. [51,52]. Our estimate falls within this typical range, indicating consistency with broader findings on WNV prevalence in mosquito populations. High viral loads detected in individual mosquito homogenates can help identify the mosquito vector species responsible for local transmission. While more field-based observations are essential for understanding the role of local mosquito species in WNV or USUV transmission, vector competence assays in controlled experimental settings can further clarify this issue [49,50].**”**

**Reviewer #3:**

**6.**I was especially impressed with the demonstrated ease and efficiency of MX for WNV and USUV and am curious as to its application on other arboviruses and arthropod species. While I believe that this manuscript provides valuable insight into a novel means of surveillance for relevant pathogens of concern, there are areas of concern in its current form. The manuscript would greatly benefit from comparison of the proposed methods to standard practices to better demonstrate its advantages. While this may be challenging, my view is that it would increase the impact of the paper if it can be done. Overall this is an interesting paper that will be of interest to the readership of PNTD. It is generally well done, and the analyses appear to have been conducted correctly.

**Response:** *Thank you for your positive comments and constructive criticism. Please refer to question 11 for our response regarding method comparisons.*

Specific comments on the manuscript follow:

**7.**Please define WNV and USUV in the abstract prior to the author summary (line 29).

**Response***: Correction has been made.*

**8.**“MX can early detect” is confusing (line 44).

**Response:** *By early detection, we meant detection before cases occur in humans or horses. This has now been clarified.*

**Line 43:** “At the crossroads between entomological and environmental surveillance, MX can detect the circulation of zoonotic pathogens in the environment before cases are observed in humans and horses, enabling the timely alerts to health policy makers, allowing them to take suitable control measures.”

**9.**Please reference Box 1 to end of first paragraph in introduction (line 69).

**Response:** *We agree, the change has been made.*

**10.**“The emergence of these two viruses always evolves towards a state of endemicity” comes across as an overestimation. Please provide references that supports this (line 123).

**Response:** *We agree that this assertion warrants further support. Europe has witnessed WNV become endemic following initial outbreaks, with recurring cases linked to local transmission cycles that persist across seasons. Over the past three decades, different strains of WNV, primarily from lineage 1 and then lineage 2, have been introduced into Italy. Epidemiological and genetic sequencing data indicate that these strains can establish, overwinter, and becoming endemic. However, they can also extinguish locally or spread over short and long distances across European and Mediterranean countries [4,5]. The introduction of WNV in the United States in 1999 further highlights the virus’s potential to persist and evolve across seasons [6]. References were added to this sentence.*

**Line 117:** “Europe and the United States have witnessed WNV become endemic following initial outbreaks, with recurring cases linked to local transmission cycles that persist across seasons [4–6] *.*”

**11.**The introduction would benefit from the inclusion of a more detailed explanation of currently early detection methods.

**Response:** *Entomological surveillance is primarily implemented worldwide as an early warning system for viral circulation and to assess the risk of human transmission. To our knowledge, all current surveillance strategies involve pooling mosquitoes by species, date, and location before extracting and detecting viral genomic RNA. This point is now addressed in the introduction with relevant references. Hall-Mendelin and colleagues were the first to propose a method in 2010 to address the limitations of the traditional pooling approach, as explained in the following paragraph outlining the origins of the MX method.*

**Line 130: “**Detecting arboviruses in field-collected mosquitoes is primarily used worldwide as an early warning system for viral circulation and to assess the risk of human transmission [20] . In most cases, arbovirus surveillance involves pooling mosquitoes by species, date, and location before extracting and detecting viral genomic RNA. For WNV surveillance, thousands to hundreds of thousands of mosquitoes are typically collected over a season, leading to pools of 50 to 200 mosquitoes per sample to minimize costs, with reported minimum infection rates ranging from less than 2% [21–24] to 10% [24,25] across studies.**”**

**12.**The manuscript would benefit from the inclusion of a more detailed explanation of the geography of the study area. Specifically, providing background about Nouvelle-Aquitaine’s prior history of mosquito or arbovirus activity would help convey to the reader how the study was being executed (line 154-155).

**Response:** *It is now clearly specified in this sentence that WNV circulation was never documented in the Nouvelle-Aquitaine region. We document here a new area of emergence for WNV. This area is described in the Material and Method section.*

**Line 155:** “We demonstrated the value of MX in July-August 2023 in Nouvelle-Aquitaine, a region in southwest France with no prior history of WNV circulation until its initial detection in 2022.”

**13.**Is it known what the stability in storing the mosquito excreta-impregnated filter paper at room temperature? Is virus degradation observed and too what degree?

**Response:** *Virus RNA preservation is central to the MX method. Our field-based data demonstrate that it is feasible to detect viruses and even generate large amplicons from samples collected and transported at room temperature for several days before processing. To further support this, we propose adding laboratory-based evidence in a Supplementary file 4 showing that virus RNA can be preserved for over one month across various storage temperatures before to be detected by RT-qPCR. Our controlled experimental design demonstrates that Flavivirus RNA remains detectable by RT-qPCR across different support materials, virus doses, temperatures, and storage durations of up to one month, even at 37°C. This finding underscores the robust preservation of viral RNA in its dried state under diverse storage conditions. With the approval of the editor and reviewers, we would like to add an author to this publication if the inclusion of this supplementary file is accepted.*

**Line 519:** “…(ii) excreta can be preserved and transported from the field to the laboratory at room temperature by regular postal mail (supplementary file 4), bringing real-time detection and genetic identification within reach;…**”**

**Line 524: “**Virus RNA preservation is central to the MX method. In a spiking and recovery experiment using Aedes flavivirus, an insect specific flavivirus with similar morphologic and genomic characteristics than WNV, we demonstrated that dried viral RNA remained detectable by RT-qPCR across varied support materials and temperatures up to one month, even at 37°C (supplementary file 4).**”**

**14.**“The presence of a low lumber”? Please confirm is lumber is correct (line 203).

**Response:** *Thank you for identifying this error. We have corrected it in the updated document.*

**15.**Please include a separate section discussing statistical tests used (line 342-344).

**Response:** *This paragraph now appears in a separated “Statistical analyses and data visualization” section.*

**16.**Please provide a justification or reasoning behind collecting from these sites at different timeframes in Figure 1 (line 356-366).

**Response:** *The MX strategy was initially deployed in areas with documented virus circulation, based on stable localization of the 3 horses diagnosed infected by WNV in 2022 and unpublished WNV seroprevalence data from horses located in Gironde county. Traps were placed in stables with horses that had an immune response to WNV. Moreover, four traps were placed near mosquito breeding sites identified by the local collectivities . These four traps (A, B, C and D) were placed between the rural/swamp area and the anthropized area. Given that (i) entomological surveillance cannot rely on symptomatic cases, (ii) mosquito infection prevalence is typically low, (iii) only a small proportion of mosquitoes can be sampled from the surrounding environment and (iv) securing trap locations with power access is often challenging, we conducted a prospective survey at a limited number of sites over a restricted period. Thus, the timeframe can be explained by the urgency of capturing mosquitoes before the peak of the transmission season, with limited resources. Some traps were subsequently moved to urban areas in Bordeaux and near a human case in the Charente-Maritime region. In other words, the selection of study sites and the collection period were determined by an opportunist approach with the resources we had available.*

**17.**There should be discussion on why no USUV RNA was detected even though it was found in excreta as a potential limitation of XM (line 399-400).

**Response:** *The detection of viral RNA in mosquito excreta without finding corresponding infected mosquitoes raises a valid question. First, we cannot entirely rule out the possibility of mosquitoes escaping during sampling or trap handling. However, we believe the more likely explanation is the presence of predatory arthropods, such as ants and spiders, using the traps as food storage sites. Ants, in particular, probably first attracted by the sugar source inside the adapters, have been observed severing mosquitoes' legs or wings and carrying them back to their nests, leaving detached mosquito parts in the collections. This recurring issue has yet to be resolved with a simple solution. Despite this challenge, the MX approach remains valuable as it increases the overall probability of detecting trapped infected mosquitoes. These limitations are now addressed in the discussion*.

**Line 597:** “In this study, as in previous one [22], viral RNA was detected in the excreta of trapped mosquitoes, even when none was found in the mosquitoes themselves. Although we cannot completely rule out the possibility of mosquitoes escaping during sampling or trap handling, the more plausible explanation is the presence of predatory arthropods, such as ants and spiders, using the traps as food storage. Ants, in particular, probably first attracted by the sugar source inside the adapters, have been observed severing mosquitoes' legs or wings and carrying them back to their nests, leaving detached mosquito parts in the collections. This recurring issue, particularly associated with long-term field captures over several days, remains difficult to resolve. Integrated approaches that leverage site-specific solutions can help mitigate this issue, such as suspending traps from a glue-covered wire or placing them over a basin of water.”

**18.**This is a great demonstration of MX use and capabilities (line 502-508).

**Response:** *Thank you for this comment* .

**19.**Have the authors completed a side-by-side comparison of detecting virus in diluted mosquito excreta to determine the level of detection?

**Response:** *The sensitivity of the MX method depends on the amount of viral genomic RNA shed at the bottom of the adapter, which in turn is influenced by the number of trapped infected mosquitoes, their viral load, and the time they spend in the adapter before their excreta are collected. This work has shown that 2 infected mosquitoes trapped during few days are sufficient to detect WNV or USUV and that Culex mosquitoes infected with WNV or USUV with Ct below 20 are regularly found in traps. Individual Aedes aegypti mosquitoes experimentally infected with dengue virus via oral exposure were reported to shed large amounts of DENV RNA in their excreta (ranging from 1.71 × 10³ to 1.24 × 10⁵ RNA copies per female per day), far exceeding the molecular detection threshold of RT-qPCR. As the reviewer suggested, further controlled experiments in a BSL-3 facility are necessary to address this question.*

**Reviewer #4:**

**20.**This is an extremely well-written, well-structured, and carefully analysed paper. MX is highly topical, as is the difficulty in defining transmission pathways for arboviral zoonoses with many vectors and reservoirs.  The authors used a nice combination of PCR, viral isolation and sequencing to deliver robust conclusions about the utility of this excreta-based, MX method. Mosquito IDs and vertebrate hosts were identified through the incorporation of conventional barcoding. The authors should be congratulated on delivery of an excellent manuscript and for making an important contribution to the field.

**Response:** *We thank the reviewer for these positive comments***.**

## References

1.	Saldanha I, Lea R, Manangwa O, Garrod G, Haines LR, Acosta-Serrano Á, et al. Caught in a trap: DNA contamination in tsetse xenomonitoring can lead to over-estimates of Trypanosoma brucei infection. PLoS Negl Trop Dis. 2024;18: e0012095. doi:10.1371/journal.pntd.0012095

2.	Andreadis TG, Anderson JF, Vossbrinck CR. Mosquito surveillance for West Nile virus in Connecticut, 2000: isolation from Culex pipiens, Cx. restuans, Cx. salinarius, and Culiseta melanura. Emerg Infect Dis. 2001;7: 670–674. doi:10.3201/eid0704.010413

3.	Johnson BJ, Munafo K, Shappell L, Tsipoura N, Robson M, Ehrenfeld J, et al. The roles of mosquito and bird communities on the prevalence of West Nile virus in urban wetland and residential habitats. Urban Ecosyst. 2012;15: 513–531. doi:10.1007/s11252-012-0248-1

4.	Barzon L, Pacenti M, Montarsi F, Fornasiero D, Gobbo F, Quaranta E, et al. Rapid spread of a new West Nile virus lineage 1 associated with increased risk of neuroinvasive disease during a large outbreak in Italy in 2022. Journal of Travel Medicine. 2022; taac125. doi:10.1093/jtm/taac125

5.	Barzon L, Montarsi F, Quaranta E, Monne I, Pacenti M, Michelutti A, et al. Early start of seasonal transmission and co-circulation of West Nile virus lineage 2 and a newly introduced lineage 1 strain, northern Italy, June 2022. Eurosurveillance. 2022;27. doi:10.2807/1560-7917.ES.2022.27.29.2200548

6.	Hadfield J, Brito AF, Swetnam DM, Vogels CBF, Tokarz RE, Andersen KG, et al. Twenty years of West Nile virus spread and evolution in the Americas visualized by Nextstrain. Hobman TC, editor. PLoS Pathog. 2019;15: e1008042. doi:10.1371/journal.ppat.1008042
